# Transcription Factor ATF4 Deletion Reprograms Glucose Metabolism in Clear Cell Renal Cell Carcinoma

**DOI:** 10.3390/cancers18121953

**Published:** 2026-06-16

**Authors:** Yuling Chi, Qiuying Chen, Eduardo Mere Del Aguila, Steven S. Gross, John A. Wagner, Shannon M. Reilly, David M. Nanus, Lorraine J. Gudas

**Affiliations:** 1 Pharmacology Department, Cornell University, 1300 York Ave, New York, NY 10065, USA; yuc4019@med.cornell.edu (Y.C.); qic2005@med.cornell.edu (Q.C.); edm4001@med.cornell.edu (E.M.D.A.); 2 Brain and Mind Research Institute, Cornell University, 1300 York Ave, New York, NY 10065, USA; jawagne@med.cornell.edu; 3 Cell and Developmental Biology, Cornell University, 1300 York Ave, New York, NY 10065, USA; 4 Department of Medicine, Cornell University, 1300 York Ave, New York, NY 10065, USA; smr4005@med.cornell.edu (S.M.R.); dnanus@med.cornell.edu (D.M.N.); 5 Weill Cornell Medicine and Weill Cornell Graduate School of Medical Sciences, Cornell University, 1300 York Ave, New York, NY 10065, USA

**Keywords:** ATF4, clear cell renal cell carcinoma (ccRCC), metabolomics, glycolysis, mitochondrial oxidative phosphorylation

## Abstract

**Background/Objectives**: Clear cell renal cell carcinoma (ccRCC) is the most common form of kidney cancer. Human ccRCCs have increased glycolytic metabolism and decreased mitochondrial oxidative metabolism relative to normal kidneys. Our research using human RCC4 ccRCC cells and a murine model of ccRCC, TRACK (TRAnsgenic model/Cancer/Kidney), in which a triple-mutant (P402A, P564A, N803A) human HIF1α is selectively expressed in proximal tubule cells (PTCs), revealed highly induced ATF4, a stress-responsive transcription factor. We then investigated the role of ATF4 in the metabolic changes in ccRCC. **Methods**: We performed comprehensive analysis of the ccRCC Cancer Genomics Atlas (TCGA) data. We deleted ATF4 in PTCs of TRACK mice and human RCC4 cells. We conducted genome-wide transcriptomic and untargeted metabolomic studies of cortices of WT and CGERA∆T (TRACK mice with PTC-specific ATF4-knockout (KO)) mice and performed glucose isotopologue tracing in parental and ATF4 KO RCC4 cells. **Results**: Analysis of TCGA data showed increased mRNAs of enzymes in glycolysis and reduced mRNAs of enzymes in the TCA cycle. Transcriptomic and metabolomic studies demonstrated that ATF4 deletion suppressed glycolysis and enhanced TCA cycle metabolism in CGERA∆T versus WT cortices. Glucose isotopologue tracing showed that ATF4 deletion altered glycolysis pathway metabolite levels and shifted glucose metabolism towards the TCA cycle, evidenced by increased intracellular [^13^C_2_]citrate in RCC4-ATF4 KO cells. Using the Seahorse XFe96 analyzer we also showed reduced glycolytic capacity and reserve in RCC4-ATF4 KO cells. **Conclusions**: Collectively, our results demonstrate that ATF4 regulates glycolysis in ccRCC, supporting ATF4 as a therapeutic target.

## Simple Summary

Clear cell renal cell carcinoma (ccRCC) is the most common form of kidney cancer, accounting for 75–85% of cancer-related deaths. ccRCC is a Von Hippel–Lindau (VHL) disease in which hypoxia-inducible factor-1α (HIF1α) and HIF2α are constitutively active, contributing to dysregulated metabolism. We engineered a mouse model of ccRCC, TRACK (TRAnsgenic model/Cancer-Kidney), with a triple-mutant human HIF1α selectively expressed in proximal tubule cells (PTCs). In determining the molecular mechanisms causing ccRCC, we found that ATF4, a stress-responsive transcription factor, and its target genes were significantly induced in TRACK mice and human ccRCC RCC4 cells. We then deleted ATF4 in PTCs of TRACK kidneys and in RCC4 cells. Using multiple cutting-edge technologies, we demonstrate that ATF4 deletion reduces glycolysis and shifts glucose metabolism to greater mitochondrial oxidative metabolism. These findings advance our understanding of the molecular causes of ccRCC and provide evidence supporting ATF4 as a therapeutic target.

## 1. Introduction

Clear cell renal cell carcinoma (ccRCC) is the most common form of kidney cancer in adults, accounting for about 75–85% of kidney cancer-related deaths [1,2,3,4]. Normal human kidneys have well established oxygen tension gradients that regulate and respond to oxygen, glucose, and other critical metabolic features to carry out the specialized functions of filtering waste products and transporting ions to maintain osmotic homeostasis [5]. In the vast majority of ccRCC patients, however, the oxygen gradient is largely disrupted and the tumor microenvironment becomes hypoxic. Biallelic loss of Von Hippel–Lindau (VHL) tumor suppressor gene expression [6] leads to abnormal, pseudohypoxic activation of hypoxia-inducible factor-1α (HIF1α) and HIF2α signaling [7,8]. This activation of HIF1α contributes to metabolic derangements, including increased lipid uptake and synthesis, enhanced glycolysis, and reduced mitochondrial oxidation [8,9,10].

We have generated a murine TRAnsgenic model of Cancer of the Kidney (TRACK) that expresses a triple-mutant (P402A, P564A, and N803A) human HIF1α construct in murine proximal tubule cells (PTCs). These mutations in the oxygen-dependent degradation domain of HIF1α prevent HIF1α recognition by VHL, interfere with the proteasomal degradation of this mutant HIF1α, and enhance its transcriptional activity [11,12]. TRACK PTCs mimic the histologic alterations found in early-stage human ccRCC even though this model does not fully recapitulate the genomic heterogeneity of human disease [12]. The major transcriptomic signatures in these TRACK PTCs also resemble those in ccRCC patients [11,13]. Additionally, we found that ATF4 (activating transcription factor 4) and its target genes are upregulated in both human ccRCC and TRACK cortices [14,15,16].

ATF4, a transcription factor induced by various types of cellular stress, is a major effector of the integrated stress response (ISR) [17]. The ISR is a signaling pathway which is activated during potentially pathological conditions, such as hypoxia, glucose deprivation, mitochondrial stress, and oncogene activation [18,19]. We previously reported an inverse correlation of ATF4 expression with survival in human ccRCC [14]. A master regulator of the cellular response to stress, ATF4 sensitivity to oxygen is the result of its interactions with the prolyl-4-hydroxylase domain 3 (PHD3) oxygen sensor [20]. Hypoxia and PHD inhibitors stabilize ATF4 [20]. ATF4 activation has been observed in melanoma cells in response to glucose deprivation [21], in *Drosophila* due to a disturbance of the mitochondrial electron transport chain (ETC) [22], and in murine kidney epithelial cells with disruption of the TCA (tricarboxylic acid) cycle [23]. Conversely, knockdown or knockout (KO) of ATF4 impairs glycolysis in cultured A549 cells [24], macrophages [25], and CD4^+^ T cells [26].

Thus far, regulation of glycolysis and mitochondrial oxidation by ATF4 has only been shown in *Drosophila* and cultured cells. Whether ATF4 regulates glycolysis and mitochondrial oxidative metabolism in kidney cells and in human ccRCC has not been explored. We generated a mouse model in which ATF4 is specifically deleted in the PTCs of TRACK mice [15]. In addition, we generated a human RCC4 cell line with ATF4 knocked out [14]. Using these tools, we demonstrate that ATF4 regulates the balance of glycolysis and mitochondrial oxidation in ccRCC. Specifically, ATF4 deletion partially reverses the increased glycolytic activities seen in ccRCC and mitigates the reduced mitochondrial oxidative activities in ccRCC.

## 2. Methods

### 2.1. Acquisition and Analysis of Gene Expression Data from the Database

We acquired The Cancer Genome Atlas Kidney Renal Clear Cell Carcinoma (TCGA-KIRC) data set from the National Cancer Institute (NCI) Genomic Data Commons (GDC) using TCGA biolinks. We analyzed mRNA expression in the kidneys of 68 normal kidneys and 470 ccRCC patients by using several programs, including Gene Set Enrichment Analysis (GSEA) for pathway analyses, Morpheus for heatmap generation, and Prism for comparisons of gene expression between individual patients and normal individuals.

### 2.2. Mice and Treatments

All animal experiments and protocols were approved by the Institutional Animal Care and Use Committees (IACUC) of the WCMC. The generation of mouse lines is described in our recent publication [15].

### 2.3. Transcriptomic Studies

The methods for transcriptomic studies were similar to the ones described in our recent publication [16]. Total RNA was extracted from half of a kidney reserved in RNALater when mice were sacrificed. Cortices of kidneys from 3 to 4 mice from each of the cohorts were dissected for mRNA-seq. 5–10 mg of each cortex section was transferred into a tube containing beads (Benchmark Scientific Prefilled Tubes for Homogenizer, Catalogue #, D1032-15, Benchmark, Sayreville NJ, USA) and a 500 μL mixture of RLT (from Qiagen RNeasy Plus Mini Kit (Cat. No. 74134), Qiagen, Germantown, MD, USA) and β-mercaptoethanol. Tissues were homogenized twice for 30 s each with a Bead Bug homogenizer (Benchmark Scientific, Fort Lauderdale, FL, USA). After homogenization, total RNA was extracted by following the protocol from the kit.

Total RNA was submitted to the WCM Genomics Resources Core. Quality control (QC) was run for each sample. Samples with RNA integrity number (RIN) ≥ 9.0 were selected and transcribed to cDNAs. Libraries (40,000,000 fragments/library) were constructed and fed into NovaSeq 6000 for sequencing with pair-end 51 bps. .

The raw sequencing reads in BCL format were processed through Illumina bcl2fastq v2.20 for demultiplexing and FASTQ conversion. Following adaptor and low-quality base trimming with Cutadapt v3.5, the reads were mapped to the GRCm38 mouse reference genome using STAR v2.7.9a [27]. Read counts per gene were extracted using HTSeq-count v0.13.5 [28]. Fragments per kilobase of transcript per million mapped reads (FPKMs) were calculated to normalize read counts. Genes with FPKM ≥ 1 were considered present and were selected for differential expression analysis between two conditions/groups. Gene differential expression analysis was performed using the DESeq2 package v1.38.3 [29]. Pathway enrichment analysis was performed with the R package clusterProfiler v4.6.2 [30], and the results were visualized using the R package ggplot2 v3.4.1.

### 2.4. Metabolomic Studies

Metabolite extraction and analysis were conducted as described [16]. Total metabolites were extracted from snap frozen kidney tissues using −70 °C 80:20 methanol:water (LC-MS grade methanol, Fisher Scientific, Waltham, MA, USA). The tissue–methanol mixture was subjected to bead-beating for 45 s using a Tissuelyser cell disrupter (Qiagen, Germantown, MD, USA). Extracts were centrifuged at 5000 rpm for 5 min to pellet insoluble protein and supernatants were transferred to clean tubes. The extraction procedure was repeated two additional times and all three supernatants were pooled, dried in a Vacufuge (Eppendorf, Hauppauge, NY, USA) and stored at −80 °C until analysis. The methanol-insoluble protein pellet was solubilized in 0.2 M NaOH at 95 °C for 20 min and protein was quantified using a BioRad DC assay. On the day of metabolite analysis, dried cell extracts were reconstituted in 70% acetonitrile at a relative protein concentration of 3.1 µg/mL, and 4 µL of this reconstituted extract was injected for LC/MS-based targeted and untargeted metabolite profiling.

Metabolite extracts from tissue were analyzed by LC/MS as described previously [31], using a platform comprising an Agilent Model 1290 Infinity II liquid chromatography system coupled to an Agilent 6550 iFunnel time-of-flight MS analyzer. Chromatography of metabolites utilized aqueous normal phase (ANP) chromatography on a Diamond Hydride column (Microsolv Technology Corporation, Eatontown, NJ, USA). Mobile phases consisted of (A) 50% isopropanol, containing 0.025% acetic acid, and (B) 90% acetonitrile containing 5 mM ammonium acetate. To eliminate the interference of metal ions with chromatographic peak integrity and electrospray ionization, EDTA was added to the mobile phase at a final concentration of 5 µM. The following gradient was applied: 0–1.0 min, 99% B; 1.0–15.0 min, to 20% B; 15.0 to 29.0, 0% B; 29.1 to 37 min, 99% B. Raw data were analyzed using MassHunter Profinder 10.0 and MassProfiler Professional (MPP) 15.1 software (Agilent Technologies, Santa Clara, CA, USA). Student *t*-tests (*p* < 0.05) were performed to identify significant differences between groups.

To ascertain the identities of differentially expressed metabolites, LC/MS data was searched against an in-house annotated personal metabolite database created using MassHunter PCDL manager 8.0 (Agilent Technologies), based on monoisotopic neutral masses (<5 ppm mass accuracy) and chromatographic retention times. A molecular formula generator (MFG) algorithm in MPP was used to generate and score empirical molecular formulae, based on a weighted consideration of monoisotopic mass accuracy, isotope abundance ratios, and spacing between isotope peaks. A tentative compound ID was assigned when PCDL database and MFG scores concurred for a given candidate molecule. Tentatively assigned molecules were verified based on a match of LC retention times and/or MS/MS fragmentation spectra for pure molecule standards contained in a growing in-house metabolite database.

### 2.5. Stable Isotope Tracing

The RCC4 line [14] was used to generate RCC4-ATF4 knockout (KO) cells. When cells (either parental (RCC4-P) or RCC4-ATF4 KO) became confluent in about 5–7 days, we seeded 0.5 × 10^6^–0.6 × 10^6^ cells per 3 cm plate. Forty-eight hours later, we washed cells with warm PBS twice, each time aspirating and removing as much PBS as possible. We then added 1 mL tracing medium (Gibco (Grnad Island, NY, USA) RPMI 1640 with glutamine, without glucose, 11879-020) containing 10% dialyzed FBS (Atlantis Bioscience (Singapore), S181D-500), and 5 mM [U^13^C_6_]D-glucose (MedChemExpress (MEC) (Monmouth Junction, NJ, USA), HY-B0389A) or 5 mM [^12^C]D-glucose, and incubated cells at 37 °C for 5, 10 or 30 min. At 5, 10 or 30 min, we took cells out of the incubator, immediately removed medium, washed cells with cold PBS twice and with cold ddH_2_O once, and aspirated and removed all residual PBS or ddH_2_O after each wash. We then immediately added 400 μL of 80% methanol, prepared one day prior and kept at −75 °C overnight. The plates were placed on ice and cells scraped off the plates and transferred into a 2.0 mL special tube (Qiagen, 1050299) containing 1 grinding ball (OPS Diagnostics (Lebanon, NJ, USA), GBSS 156-5000-01). The plates were rinsed with 150 μL cold 80% methanol twice, combining all 80% methanol mixtures in the same tube. The tubes were kept at −20 °C for 10 min and metabolites were extracted or the samples were kept at −80 °C and metabolites extracted later.

### 2.6. Extracellular Acidification Rate (ECAR) and Oxygen Consumption Rate (OCR) Measurements

Fifty thousand RCC4-P or RCC4-ATF4 KO cells were seeded on a Seahorse XFe96 96-well microplate. Twenty-four hours later, the media was changed to bicarbonate free media and the cells were incubated at 37 °C, without CO_2_, for 90 min. For the glucose stress test, the new medium did not contain glucose. Baseline ECAR was recorded for 20 min. Thereafter, 5 mM (final concentration) glucose, 1.2 μM (final concentration) oligomycin, and 50 mM (final concentration) 2-deoxy-D-glucose (2DG) were injected sequentially, at indicated times, through ports A, B, and C, respectively. For the mitochondrial stress test, the new medium contained 5 mM glucose. Baseline OCR was recorded for 20 min. Thereafter, 2 μM oligomycin, 1 μM carbonyl cyanide 4-(trifluoromethoxy) phenylhydrazone (FCCP), and 1 μM rotenone and 1 μM antimycin A together were injected sequentially at the indicated times through ports A, B, and C, respectively. Time courses of ECAR and OCR were normalized to amount of protein/well. Glycolytic capacity was calculated as the difference between ECAR following the injection of 1 µm oligomycin and the basal ECAR reading. Glycolytic reserve was calculated as the difference in ECAR between glucose and oligomycin injections. For OCR, total respiration capacity was calculated as the total area under the curve (AUC) according to the protocol in the Agilent Seahorse XF Cell Mito Stress Test Kit. These experiments were conducted three times, each time with 8 replicates for each condition.

### 2.7. Statistics

Statistical analysis was performed using GraphPad Prism 10.4.2. Data are expressed as the mean ± SD. Statistical differences between 2 groups were analyzed by unpaired, 2-tailed Student’s *t* test. For multiple group comparisons, we used Prism and chose One-way ANOVA. The sample size was determined based on our prior experiments in the lab. A *p* value of less than 0.05 was considered as statistically significant.

## 3. Results

### 3.1. Upregulated Glycolysis and Downregulated Mitochondrial Transcripts in Human ccRCCs

**Figure 1:**
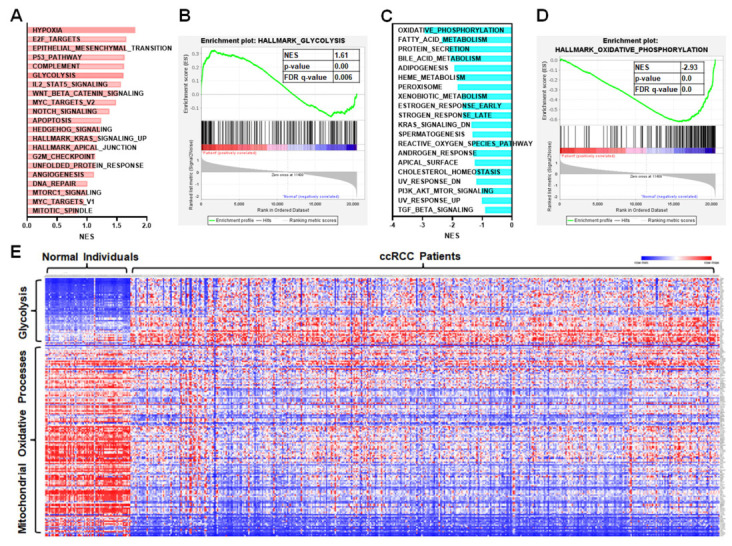
Upregulated glycolysis and downregulated mitochondrial transcripts in human ccRCCs. GSEA of transcripts involved in glycolysis and mitochondrial oxidative activities in 470 ccRCC patient samples and 68 normal kidney samples. (**A**) Top 20 upregulated gene sets in ccRCCs compared to normal kidney samples; (**B**) gene enrichment plot of glycolysis; (**C**) top 20 downregulated gene sets in ccRCCs compared to normal kidney samples; (**D**) gene enrichment plot of oxidative phosphorylation; (**E**) heatmap of transcripts involved in glycolysis and mitochondrial oxidative activities. NES, Normalized Enrichment Score.

Since the landmark report of the Cancer Genome Atlas (TCGA) on ccRCC [32], several studies have demonstrated dysregulated glycolysis and the TCA cycle activity in human ccRCC [33]. Most of these reports focused on correlations of survival rate or stages of ccRCC with gene expression pattens [32,34,35,36], or focused on specific aspects of ccRCC, such as immune scores [37], T-cell immune regulator 1 (TCIRG1) [38], or long non-coding RNAs (lncRNAs) [39,40]. There has not been a detailed and complete genome-wide analysis of individual genes and pathways of genes participating in both glycolysis and the TCA cycle. To do this analysis we downloaded the ccRCC Cancer Genomics Atlas repository (TCGA) data from the National Cancer Institute (NCI) Genomic Data Commons (GDC) using TCGA biolinks and analyzed mRNA levels in 68 normal human kidneys and 470 human ccRCC samples. GSEA revealed glycolysis as the 6th top upregulated gene set in ccRCC patients compared to normal kidneys (Figure 1A,B), while mitochondrial oxidative phosphorylation was the most downregulated gene set (Figure 1C,D). A heatmap of genes involved in these two processes clearly shows well-clustered overexpression of genes involved in glycolysis and a reduction in expression of genes participating in mitochondrial oxidative activities (Figure 1E).

**Figure 2:**
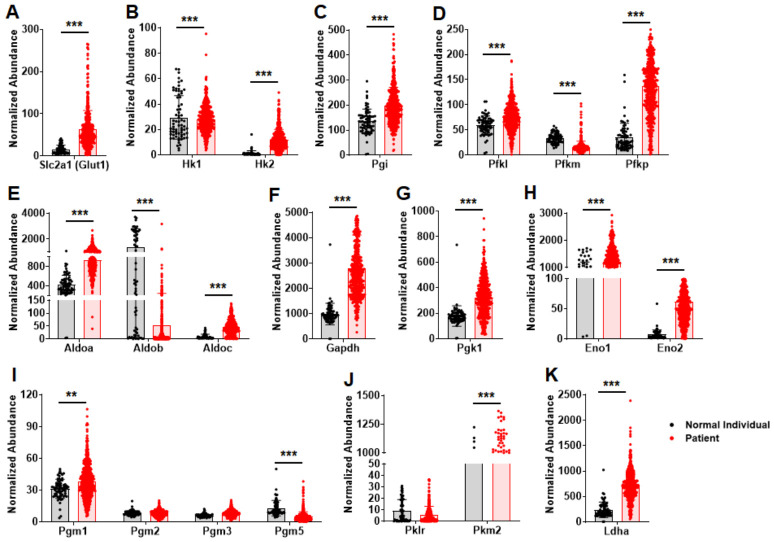
Induced transcripts in glycolysis pathway in ccRCC samples. Values are normalized to mRNA abundance obtained by mRNA sequencing of tumor samples from 470 ccRCC patients and 68 normal kidney samples. (**A**–**K**), bar graph of expressions of gene indicated on x-axis. Value = average ± std (standard deviation).** *p* < 0.01, *** *p* < 0.001. Slc2a1 (Glut1), solute carrier family 2 member 1 (glucose transporter member 1); Hk: hexokinase; Pgi, phosphoglucose isomerase; Pfkl, phosphofructokinase, liver; pfkm, phosphofructokinase, muscle; Pfkp, phosphofructokinase, platelet; Aldo, aldolase; Gapdh, glyceraldehyde-3-phosphate dehydrogenase; Pgk1, phosphoglycerate kinase 1; Eno, enolase; Pgm, phosphoglucomutase; Pklr, pyruvate kinase L/R; Pkm2, pyruvate kinase M2; Ldha, lactate dehydrogenase a.

A more detailed analysis of genes involved in glycolysis is illustrated in Figure 2. Glycolysis starts with glucose uptake mediated by SLC2A1 (GLUT1) [41]. Expression of *SLC2A1* mRNA increased by more than 4-fold in ccRCC compared to normal kidneys (Figure 2A). Intracellular glucose is then metabolized to glucose-6-phosphate by hexokinase (HK) [42,43,44]. Two *HK*s are detected, *HK1* and *HK2*. While the expression of *HK1* mRNA in ccRCC was similar to that in normal kidneys, *HK2* mRNA was more than 10-fold higher in ccRCC than in normal kidneys (Figure 2B). Glucose-6-phosphate (G6P) is converted to fructose-6-phosphate (F6P) by phosphoglucose isomerase (*PGI*), also called glucose-6-phosphate isomerase (*GPI*), which was also higher in ccRCC than in normal kidneys (Figure 2C). The next step, conversion of F6P to fructose-1,6-bisphosphate (FBP), another regulatory step in glycolysis, is catalyzed by phosphofructokinase (PFK). There are three isozymes of *PFK*, *PFKL* (liver), *PFKM* (muscle) and *PFKP* (pancreas) [45]. All three isozymes are expressed in the kidney. *PFKL* is the most abundant isozyme in normal kidneys and its mRNA is slightly higher in ccRCC than in normal kidneys. *PFKM* is reduced in ccRCC, whereas *PFKP* mRNA is almost 5-fold higher in ccRCC than in normal kidneys (Figure 2D).

The following steps in glycolysis are reversible reactions catalyzed by aldolases (ALDOA and ALDOB), glyceraldehyde-3-phosphate dehydrogenase (GAPDH), phosphoglycerate kinase (PGK), phosphoglucomutases (PGM1–3, 5), enolases (ENO1 and ENO2), and pyruvate kinases (PKLR and PKM) [46,47]. Most of these transcripts are abundantly expressed and these mRNAs are higher in ccRCCs than in normal kidneys (Figure 2E–J). Lactate dehydrogenase (LDHA) catalyzes the conversion of pyruvate to lactate at the end of glycolysis and its mRNA is almost 3-fold higher in ccRCC than in normal kidneys. Thus, transcripts encoding the majority of enzymes catalyzing glycolysis are highly elevated in human ccRCCs compared to normal kidneys. Most importantly, transcripts for the three enzymes *SLC2A1*, *HK2*, and *PFK*, which catalyze the early, regulatory steps of glycolysis [43], are massively increased in ccRCC. These enzymes are also considered to be markers of ccRCC and are used in clinical settings [36,48].

**Figure 3:**
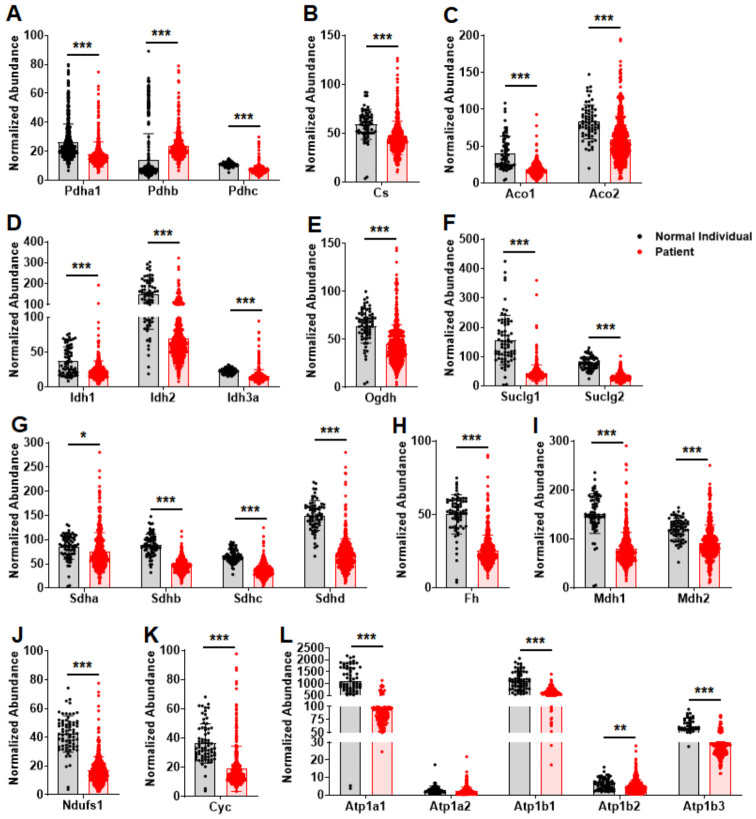
Suppressed transcripts in mitochondrial oxidation in ccRCCs. Values are normalized to mRNA abundance obtained by mRNA sequencing of tumor samples from 470 ccRCC patients and 68 normal kidney samples. (**A**–**L**), bar graph of expressions of gene indicated on x-axis. Value = average ± std (standard deviation). * *p* < 0.05, ** *p* < 0.01, *** *p* < 0.001. Pdha1, pyruvate dehydrogenase E1 subunit alpha 1; Pdhb, pyruvate dehydrogenase E1 subunit beta; Cs, citrate synthase; Aco1, aconitase 1; Idh1, isocitrate dehydrogenase 1; Ogdh, oxoglutarate dehydrogenase; Suclg1, succinate-CoA ligase GDP/ADP-forming subunit alpha; Suclg2, succinate-CoA ligase GDP/ADP-forming subunit beta; Sdha, succinate dehydrogenase complex flavoprotein subunit a; Sdhb, succinate dehydrogenase complex iron sulfur subunit b; Sdhc, succinate dehydrogenase complex subunit c; Sdhd, succinate dehydrogenase complex subunit d; Fh, fumarate hydratase; Mdh, malate dehydrogenase; Ndufs1, NADH:ubiquinone oxidoreductase core subunit s1; Cyc, cytochrome C; Atp1a, ATPase Na^+^/K^+^ transporting subunit alpha; Atp1b, ATPase Na^+^/K^+^ transporting subunit beta.

While transcripts encoding glycolytic enzymes are upregulated in ccRCC, transcripts encoding enzymes that carry out the mitochondrial TCA cycle reactions and electric transport chain (ETC) activities are generally lower (Figure 3). Pyruvate is a divergent point for glucose metabolism [49]. It can be converted to lactate or enter the mitochondria to be converted to acetyl-CoA and used to fuel the TCA cycle and ETC [49]. Transcripts encoding the enzymes in the TCA cycle are reduced in ccRCC by 2–8-fold compared to levels in normal kidneys (Figure 3A–H). Three of these enzymes, CS, IDH, and OGDH, catalyze irreversible steps, and hence decisively propel the cycle to produce succinyl-CoA and subsequently succinate [50]. The mRNAs for the most abundantly expressed isoforms (*IDH1* and *IDH2*) are reduced by 4-fold in ccRCC compared to normal kidneys. SDH is a unique enzyme in that it participates in both the TCA cycle and the ETC [51,52,53]. Transcripts for all isozymes of *SDH* are expressed at much lower levels in ccRCC than in normal kidneys (Figure 3G). Besides SDH (in complex II), mRNAs of proteins in other complexes in the ETC are also lower in ccRCCs compared to normal kidneys. For instance, the mRNAs of *NDUFS1* (the largest subunit of complex I [54]) and cytochrome c (Cyc) (complex IV) in ccRCCs are only 50% of those in normal kidneys (Figure 3J,K). Some isoforms of *ATP1* are drastically reduced in ccRCCs compared to normal kidneys (Figure 3L). Together, the data shown (Figure 2 and Figure 3) strongly suggest that glycolysis is increased in human ccRCCs compared to normal kidneys while mitochondrial oxidative capability is decreased.

### 3.2. Transcripts Encoding Enzymes in Glycolysis Are Upregulated in TRACK Cortices

**Figure 4:**
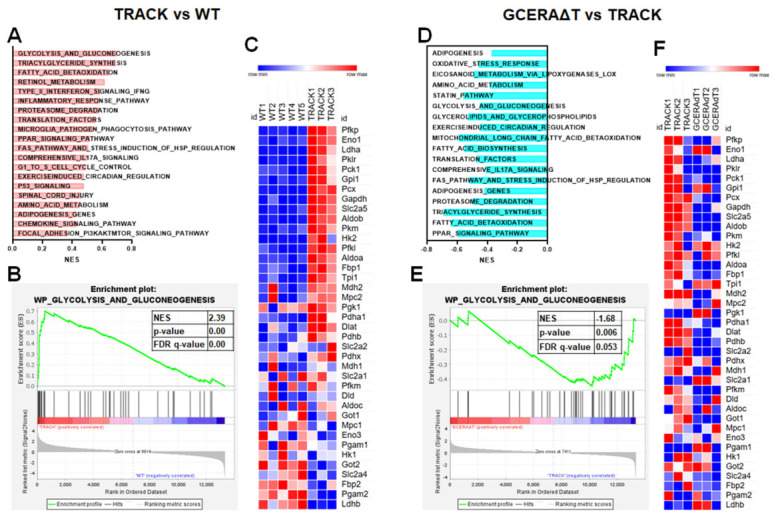
ATF4 deletion blunts the HIF1α-associated glycolytic metabolism in TRACK kidneys. GSEA of mRNA-seq data showing (**A**) upregulated top 20 gene sets in TRACK compared to WT; (**B**) enrichment plot of genes in glycolytic metabolism in TRACK vs. WT; (**C**) heatmap of significantly induced genes in glycolytic metabolism in TRACK vs. WT; (**D**) downregulated top 20 gene sets in GCERAΔT compared to TRACK; (**E**) enrichment plot of genes in glycolytic metabolism in GCERAΔT vs. TRACK; (**F**) heatmap of significantly suppressed genes in glycolytic metabolism in GCERAΔT vs. TRACK. NES, Normalized Enrichment Score.

The murine TRACK model that we generated mimics the early stage of human ccRCC [12]. To investigate whether glycolysis is altered in the TRACK kidneys, we conducted a genome-wide transcriptomics study. GSEA shows that glycolysis is the most upregulated gene set in TRACK cortices (Figure 4A,B). Similar to the changes detected in human ccRCC (Figure 2), heatmaps show close clustering of glycolysis genes and increases in almost all transcripts in the glycolysis pathway (Figure 4C). Most transcripts are increased by 2–5-fold in TRACK vs. wild type (WT) kidney cortices. Detailed analyses of individual mRNAs encoding the enzymes in glycolysis are shown (Supplementary Figure S1). These alterations in glycolysis in TRACK cortices mimic those observed in tumors of ccRCC patients. Thus, TRACK mice serve as a useful model for investigation of the causes of abnormal glucose metabolism in ccRCC.

### 3.3. ATF4 Deletion Reduces Transcripts Encoding Enzymes in Glycolysis in TRACK Cortices

Our previously reported data analysis of the TCGA-KIRC revealed upregulation of ATF4 and its target genes in human ccRCCs compared to normal kidney samples [14]. ATF4 regulation of glucose metabolism has been shown in WT mice [55]. However, whether or not ATF4 plays any role in glycolysis in the context of ccRCC is unknown. To delineate the role of ATF4 in ccRCC we generated a mouse line (GCERA∆T) with ATF4 specifically deleted in the proximal tubule cells (PTCs) of TRACK mice by a brief tamoxifen treatment in adult mice [15]. The effects of ATF4 deletion on renal physiology and pathology have been reported in our recent publication [15] in which we demonstrated massive lipid accumulation in TRACK kidneys, which is the most dominant pathological feature of ccRCC, and, importantly, ATF4 deletion mitigated lipid accumulation by inhibiting fatty acid and lipid syntheses [15].

Here, we show that glycolysis is one of the top downregulated gene sets in GCERA∆T vs. TRACK kidney cortices revealed by GSEA of genome-wide mRNA-seq of cortices (Figure 4D,E). Heatmaps show that almost all of the transcripts involved in glycolysis that are high in TRACK vs. WT cortices are lower in GCERA∆T vs. TRACK cortices (Figure 4F). These data indicate that ATF4 deletion in GCERA∆T, to a large extent, can reverse the abnormally high expression of glycolytic transcripts in the TRACK model of early ccRCC. For example, ATF4 deletion reverses the increases in the two most abundantly expressed mRNAs, *Aldob* and *Gapdh*, in the glycolysis pathway in the TRACK model (Supplementary Figure S1).

### 3.4. ATF4 Deletion Alters Metabolite Profiles in TRACK Kidney Cortices

**Figure 5:**
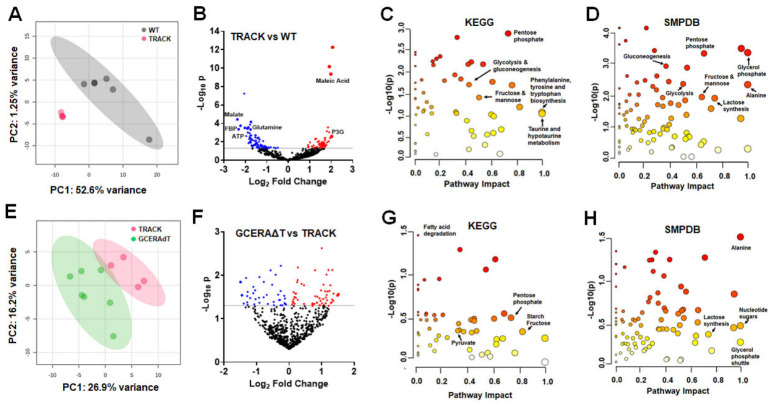
ATF4 deletion in the proximal tubules alters metabolite profiles in TRACK kidneys. (**A**,**E**) Principal component analysis (PCA) of metabolites in kidney cortices of TRACK vs. WT mice (**A**) and GCERAΔT vs. TRACK (**E**); (**B**,**F**) volcano plots of −log10(padj) versus log2(fold change) of metabolites of TRACK over WT (**B**) and GCERAΔT over TRACK (**F**). Significantly reduced metabolites are in blue; significantly increased metabolites are in red. (**C**,**G**) Kyoto Encyclopedia of Genes and Genomes (KEGG) pathways of metabolites in kidney generated by MetaboAnalyst for comparison of TRACK over WT (**C**) and GCERAΔT over TRACK (**G**); (**D**,**H**) small molecule pathway data base (SMPDB) pathways of metabolites in kidney generated by MetaboAnalyst for comparison of TRACK over WT (**D**) and GCERAΔT over TRACK (**H**). Each dot in Figures (**A**,**E**) represents a mouse.

Furthermore, we measured the levels of glycolytic metabolites, a more direct assessment of this metabolic process. To determine whether ATF4 deletion affects metabolic profiles we conducted untargeted metabolomic studies of the cortices of three groups of mice, WT, TRACK, and GCERA∆T. Six hundred and fifty-two metabolites were detected in all three groups. Principal component analysis (PCA) shows that metabolites in TRACK and WT are closely clustered and well separated (Figure 5A), indicating that the metabolic profiles are largely different between these two groups of mice. Twenty-one percent of metabolites were significantly changed in TRACK cortices compared to WT, among which 42% were upregulated and 58% were downregulated (Figure 5B). Those changes impacted several pathways. The pathways consistently implicated by both KEGG and SMPDB analyses are carbohydrate metabolism pathways, including metabolism of glucose, fructose and pentose (Figure 5C,D), consistent with a recent report on metabolic changes in human ccRCC [56]. These data, together with the mRNA-seq data, demonstrate that the metabolic status of glycolysis in TRACK mice, to a large extent, mimics that in ccRCC patients.

Next, we turned to the effects of ATF4 deletion on glycolysis in TRACK mice. The differential analyses of GCERA∆T over TRACK metabolites are shown (Figure 5E–G). PCA shows that the GCERA∆T group is separated from TRACK (Figure 5E). Fourteen percent of metabolites were significantly changed in GCERA∆T vs. TRACK (Figure 5F), and the changes also occurred primarily in carbohydrate metabolism (Figure 5G,H).

**Figure 6:**
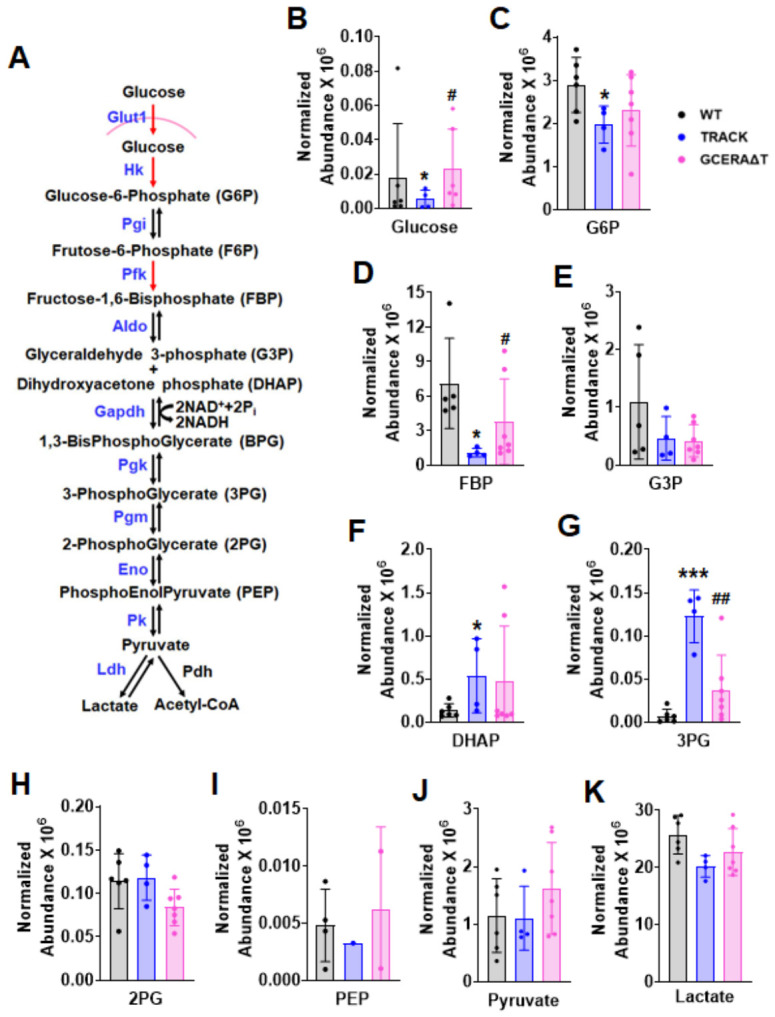
ATF4 deletion in the proximal tubules modulates metabolites in glycolysis in TRACK kidneys. (**A**), diagram of glycolysis; (**B**–**K**), bar graph of levels of metabolite indicated on x-axis. G6P, glucose 6-phosphate; FBP, fructose 1,6-bisphosphate; G3P, glyceraldehyde 3-phosphate; DHAP, dihydroxyacetone phosphate; 3PG, 3-phosphoglyceric acid; 2PG, 2-Phosphoglyceric acid; PEP, phosphoenolpyruvate. Value = average ± std (standard deviation). For comparison between TRACK vs. WT, * *p* ≤ 0.05; *** *p* ≤ 0.001; for comparison between GCERAΔT vs. TRACK, # *p* ≤ 0.05; ## *p* ≤ 0.01.

Glycolysis is illustrated in Figure 6A. The levels of the products of the early steps of glycolysis (glucose, glucose 6-phosphate (G6P) and fructose 1,6-bisphosphate (FBP)) are lower in TRACK cortices compared to WT (Figure 6B-D). Meanwhile, dihydroxyacetone phosphate (DHAP) and 3-phosphoglyceric acid (3PG) are higher in TRACK cortices compared to WT (Figure 6F,G). One of the main reasons causing lower levels of metabolites at early steps of glycolysis in TRACK cortices compared to WT cortices is dramatically upregulated *Hk2*, *Pfkp* and *Pfkl* in TRACK kidneys (Figure 4C, Supplementary Figure S1), which pull G6P forward and convert FBP into downstream products, preventing early metabolite pools from becoming larger. This is a typical phenomenon in glycolysis in tumors [57]. There are no significant differences in the remainder of the metabolites between TRACK and WT (Figure 6,E,H-K–L).

ATF4 deletion significantly altered several steps in glycolysis, resulting in increases in glucose and FBP and a decrease in 3PG in GCERA∆T cortices compared to TRACK cortices (Figure 6B,D,G). FBP is metabolized directly by Aldolase (ALDOA/B) into DHAP and GA3P, which are then further metabolized by GAPDH and BPG to 3PG. Because both *Aldoa/b* and *Gapdh* expression was profoundly decreased in GCERA∆T (Figure 4F, Supplementary Figure S1), the proximal tubules, to a great extent, have lost their capacity to metabolize FBP into metabolites of later stages of glycolysis, resulting in the accumulation of FBP and a reduction in 3PG.

**Figure 7:**
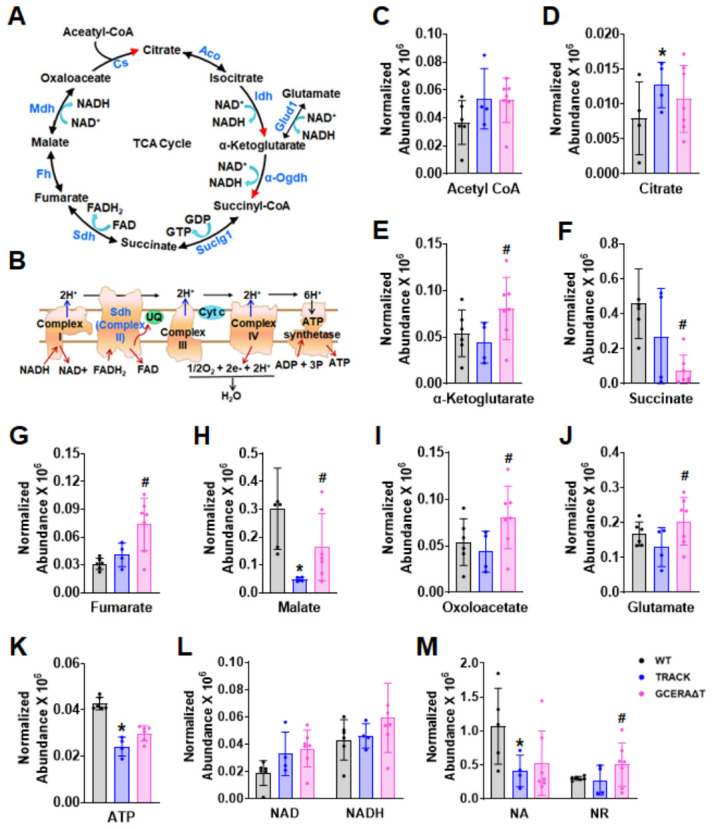
ATF4 deletion in the proximal tubules modulates metabolites in mitochondrial oxidation in TRACK kidneys. (**A**,**B**), diagrams of the TCA cycle and the ETC, respectively; (**C**–**M**), bar graph of levels of metabolite indicated at x-axis. Value = average ± std (standard deviation). For comparison between TRACK vs. WT, * *p* ≤ 0.05; for comparison between GCERAΔT vs. TRACK, # *p* ≤ 0.05.

ATF4 deletion also altered metabolites in the TCA cycle and the ETC, as illustrated in Figure 7A and B, respectively. Most metabolites in the TCA cycle, except citrate, were reduced in TRACK compared to WT cortices (Figure 7C–H), possibly resulting in a lower ATP level in TRACK compared to WT cortices (Figure 7I). Importantly, ATF4 deletion significantly increased five out of eight metabolites in the TCA cycle in GCERA∆T compared to TRACK cortices (Figure 7F–J). ATP trended higher in GCERA∆T compared to TRACK cortices (Figure 7K). Thus, the overall trend in our mouse model is that ATF4 deletion partially reverses the increased glycolysis and the reduced oxidative TCA cycle and energy production in TRACK kidney cortices.

### 3.5. ATF4 Deletion Reduces Glycolytic Metabolism in RCC4 Cells

**Figure 8:**
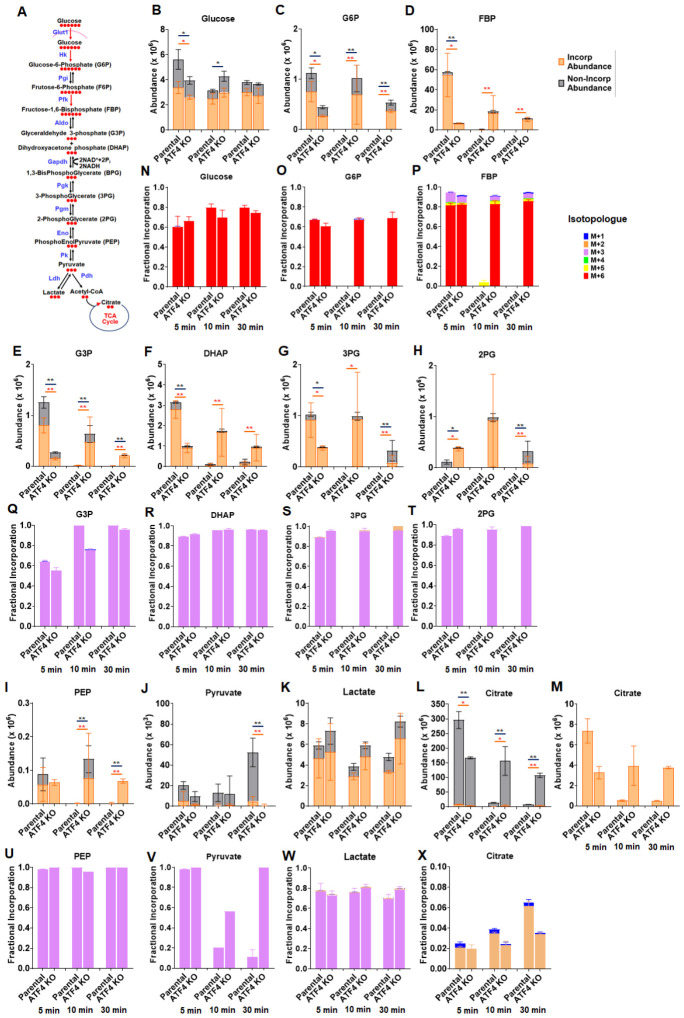
ATF4 deletion in RCC4 cells inhibits glycolytic metabolism. (**A**) Diagram of uptake and metabolism of [U^13^C_6_]glucose, red arrows are irreversible steps, black arrows are reversible steps; (**B**–**L**) abundances of metabolites incorporated or non-incorporated with ^13^C isotopes at 5, 10 or 30 min after cells were fed with [U^13^C_6_]glucose; (**M**) abundance of citrate incorporated with ^13^C isotopes; (**N**–**X**) fractional incorporations corresponding to abundances in (**B**–**L**). G6P, glucose 6-phosphate; FBP, fructose 1,6-bisphosphate; G3P, glyceraldehyde 3-phosphate; DHAP, dihydroxyacetone phosphate; 3PG, 3-phosphoglyceric acid; 2PG, 2-Phosphoglyceric acid; PEP, phosphoenolpyruvate. Value = average ± std (standard deviation). n = 3, * *p* ≤ 0.05; ** *p* ≤ 0.01. Orange asterisks indicate significance for comparisons of abundances incorporated with ^13^C isotopes between parental and ATF4KO cells; black asterisks indicate significance for comparisons of abundances non-incorporated with ^13^C isotopes between parental and ATF4KO cells.

To further test if ATF4 modifies glycolysis and oxidative phosphorylation in human cells, we conducted glucose metabolism tracing studies in RCC4-P cells (a cultured human parental ccRCC cell line), and RCC4-ATF4 KO cells [14]. We added glucose with ^13^C labeled at all six carbons to both cell types, and stopped metabolism at three time points, 5, 10, and 30 min. The fully labeled 6-carbon metabolites become fully labeled 3-carbon metabolites (Figure 8A). The abundances of all metabolites with incorporated and non-incorporated isotopes are plotted (Figure 8B–M), while the corresponding isotopologues of each metabolite are plotted in (Figure 8N–X). All metabolites with incorporated isotopes were fully labeled (Figure 8N–X). As time went on, glucose was rapidly metabolized and fluxed to the end product of glycolysis in parental RCC4 cells. ATF4 deletion effectively reduced the glycolysis flux, evidenced by accumulation of metabolites from glucose to phosphoenolpyruvate (PEP). This effect was most profound at 10 min. The levels of pyruvate are much lower compared to other metabolites, probably the result of rapid metabolism to either lactate or acetyl-CoA that subsequently enters the mitochondria and is metabolized to citrate. Although lactate levels appear higher in ATF4 KO cells, the differences are not significant. What was significant, however, were the much higher levels of citrate in ATF4 KO cells compared to the RCC4-P cells at 10 and 30 min. The majority of citrate was found as non-incorporated isotopes (Figure 8L). The zoomed in figure (Figure 8M) shows the abundance of citrate with isotopes incorporated. At 10 and 30 min, the isotope-labeled citrate species were more than 8-fold higher in ATF4 KO cells than in RCC4-P cells. Citrate has six carbons, two of which are from acetyl-CoA (from pyruvate). Almost all isotope-labeled citrate was labeled at two carbons (Figure 8X), indicating that the isotope-labeled citrate was from exogenously added [U^13^C_6_]D-glucose. These results demonstrate that ATF4 deletion in RCC4 cells provided an additional pathway for glucose metabolism toward mitochondrial oxidation metabolism.

We subsequently investigated the impact of ATF4 deletion on mitochondrial oxidation. Analyses of the metabolites in the TCA cycle at both 10 and 30 min showed that, except for citrate, all of the metabolites in the TCA cycle were not isotope-labeled in RCC4-P cells (Supplementary Figure S2). ATF4 deletion caused a 2–36 fold increase in all metabolites in the TCA cycle in RCC4 ATF4 KO cells (Supplementary Figure S2). Consequently, we detected increased production of ATP by 2–60 fold, both unlabeled and labeled with isotopes (Supplementary Figure S3F,G). Interestingly, at 30 min, 90% of isotope-labeled ATP had five carbons labeled, suggesting that this portion of ATP was synthesized from the substrate metabolized from exogenously added [U^13^C_6_]D-glucose. ATP is synthesized from ribose 5-phosphate, which is a metabolite from glucose via the pentose phosphate pathway (Supplementary Figure S3A). As time went on, ATP synthesized from glucose dramatically increased in RCC4 ATF4 KO cells compared to RCC4-P cells (Supplementary Figure S3H). All of these data demonstrate that ATF4 deletion enhances mitochondrial oxidation and energy production.

### 3.6. ATF4 Deletion Suppresses Glycolytic Capability in RCC4 Cells

**Figure 9:**
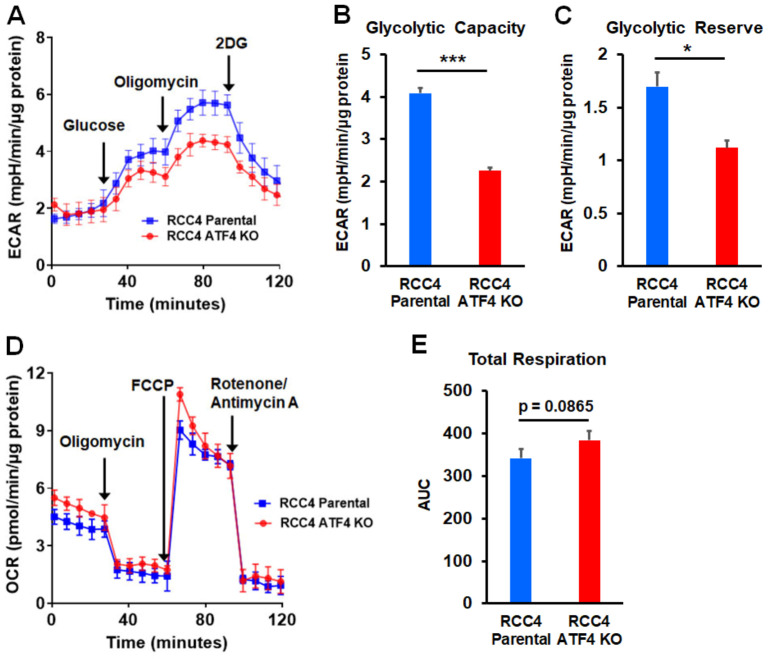
ATF4 deletion in RCC4 cells suppresses glycolytic capability. Extracellular acidification rate (ECAR) in glycolytic stress test (**A**–**C**) and oxygen consumption rate (OCR) in mitochondrial stress test (**D**,**E**) by Seahorse XFe96 Analyzer in parental RCC4 and RCC4 ATF4 KO cells. (**A**) Time course of ECAR. Cells were starved for 90 min, then baseline was run for 20 min, 5 mM glucose was injected through port 1, 2 μM oligomycin through port 2, 50 mM 2-deoxyglucose (2DG) through port 3 at the indicated time. (**B**) Glycolytic capacity calculated as the difference between ECAR following the injection of 1 μm oligomycin and the basal ECAR reading; (**C**) glycolytic reserve as the difference in ECAR between glucose and oligomycin injections. (**D**) Time course of OCR. Cells were in medium containing 5 mM glucose. After baseline data collection, 2 μM oligomycin, 1 μM carbonyl cyanide 4-(trifluoromethoxy) phenylhydrazone (FCCP), 1 μM rotenone and 1 μM antimycin A together was injected through port 1, 2, 3 separately and sequentially at the indicated time. (**E**) Total respiration capacity calculated as area under curve (AUC). Three sets of experiments were conducted for each test. For each set, there were 8 replicates under each condition. Average value of the 8 replicates was calculated for each set first. Then the average value of three sets was calculated. Error = std (standard deviation). * *p* ≤ 0.05; *** *p* ≤ 0.001.

To confirm the effects of ATF4 deletion on glucose metabolism, we conducted two sets of experiments using an Agilent XFe96 Analyzer. The first was a glucose stress test. We loaded cells onto the XFe96 cell culture microplate in medium that did not contain glucose. After initiation, 5 mM glucose was injected into the plate from port A, followed by injections of oligomycin through port B to block mitochondrial complex V and consequently to obtain maximum extracellular acidification. The last injection was 50 mM 2-deoxy-D-glucose (2-DG) through port C to eliminate glucose metabolism (Figure 9A). ATF4 deletion reduced glycolytic capacity and glycolytic reserve by 45% and 33%, respectively (Figure 9B,C).

The second set was a mitochondrial stress test measuring mitochondrial oxidation capacity. The timing of the injections of reagents is shown (Figure 9D). ATF4 deletion slightly increased total respiration (*p* = 0.086) (Figure 9E), supporting our suggestion that ATF4 modulates flux of metabolites in these pathways.

## 4. Discussion

The metabolic switch from aerobic to anaerobic metabolism, e.g., in glycolysis, is a well-accepted hallmark of cancer [58,59]. Several studies have demonstrated upregulated glycolytic processes and downregulated oxidative processes in ccRCC at the mRNA and metabolite levels [9,32,56,60]. Utilizing multi-omic approaches, here we show that in kidneys of TRACK mice, a mouse model of early ccRCC, glycolysis is enhanced while mitochondrial oxidative metabolism is reduced (Figure 4, Figure 5, Figure 6 and Figure 7), as in human ccRCC (Figure 1, Figure 2 and Figure 3) [9,32,56,60]. Importantly, we demonstrate that ATF4 deletion can partially reverse this increased glycolysis in kidneys of TRACK mice. Furthermore, ATF4 deletion reduces glycolytic metabolism and enhances mitochondrial oxidation in human RCC4 cells (Figure 8 and Figure 9, Supplementary Figures S2 and S3).

ATF4 regulation of glucose metabolism has been shown in several cell types. Knocking out ATF4 resulted in a reduction in the expression of most enzymes in glycolysis in osteoblasts and macrophages [25,55]. ATF4 knockdown in macrophages [25] and ATF4 knockout in CD4^+^ T cells [26] impair glycolytic activities. Conversely, ATF4 also promotes glycolysis which is needed for CD4^+^ T-cell immune responses [26]. Here, for the first time, we report that ATF4 deletion in kidney proximal tubules reduces glycolysis in a relevant murine carcinogenesis model. In human RCC4 cells, our glucose isotopic tracings show that ATF4 deletion blocked glycolysis flux, resulting in accumulation of most metabolites in glycolysis (Figure 8).

The increased lactate in ATF4 KO cells (Figure 8L), albeit insignificant, was unexpected. Lactate is the end product of anaerobic glucose metabolism [61]. Rapid glucose catabolism to lactate was the first molecular phenotype assigned to cancer [58,62]. However, lactate is not increased in TRACK mice (Figure 6L). Similar results were observed in ccRCC patients [9]. Lactate is another major source of energy [63]. On a molar basis, the circulatory turnover flux of lactate is the highest of all metabolites and exceeds that of glucose in fasting mice [63]. The utilization of lactate as a fuel is enhanced in tumors, such as in human lung and breast cancers [63,64,65]. One reason for the enhanced utilization of lactate as a fuel in tumors is that it circumvents the reduced glucose oxidation and supports biomass and energy production in tumors [63,64,65]. It is possible that more lactate is used as an energy source in RCC4-P cells than in ATF4 KO cells.

In ccRCC, although glycolysis is upregulated, glucose oxidation is reduced, as demonstrated by isotope tracing in human ccRCCs [9]. The main cause of the decreased glucose oxidation is the impaired mitochondrial oxidative functions carried out by the enzymes in the TCA cycle and the ETC. Transcripts of all the enzymes in the TCA cycle are significantly reduced in ccRCCs (Figure 3). The activities of those enzymes can be assessed in part by metabolite levels. Hakimi et al. showed markedly elevated citrate, cis-aconitate, and succinate, and markedly reduced fumarate and malate in ccRCCs [56]. In the report by Courtney et al. [9], citrate was not different between ccRCCs and normal kidneys. Succinate, fumarate, and malate were reduced in ccRCCs compared to normal kidneys [9]. Here we show increased citrate and reduced succinate and malate in TRACK compared to WT kidneys (Figure 7D,F,H). Considering all of these studies, the most consistent change in ccRCC is reduced malate, the metabolite at the late stage of the TCA cycle. ATF4 deletion in GCREA∆T compared to TRACK cortices almost quadrupled the malate level (Figure 7H). Elevations of metabolites in the TCA cycle upon ATF4 deletion are more impressive in RCC4 cells, in which ATF4 deletion increased the levels of all metabolites in the TCA cycle (Supplementary Figure S2), strongly suggesting an enhanced TCA cycle flux with ATF4 deletion. In addition, ATF4 deletion increased the level of glutamate, which can be converted to α-ketoglutarate in the TCA cycle (Figure 7A), in both GCERA∆T cortices (compared to TRACK cortices) (Figure 7J) and RCC4-ATF4 KO cells (Supplementary Figure S2F), providing an additional metabolite source for the mitochondrial oxidative process. The elevated level of glutamate in RCC4 ATF4 KO cells (Supplementary Figure S2F) is probably due to the increased glutamate uptake by the glutamate transporter Slc7a13, the level of which is greatly increased, as we demonstrated [16].

ATF4 involvement in the TCA cycle has been reported in cultured cells, including HeLa [66] and kidney epithelial cells [23]. Recently Labbé et al. generated a U2OS-derived cell line stably expressing dimerizable PERK [67]. Using this cell line, they showed that ATF4 deletion increased several metabolites in the TCA cycle, including citrate, acetyl-CoA, α-ketoglutarate, and malate. In HeLa cells, deficiency in ATF4 increased ATP-dependent respiration [66]. A novel finding of our study is that ATF4 deletion upregulates the TCA cycle and the ETC both in an animal model of ccRCC and in human RCC4 ccRCC cells.

The ultimate measure of mitochondrial oxidative activity is ATP production, which is lower in TRACK compared to WT kidneys (Figure 7K). ATF4 deletion increased ATP in GCERA∆T kidneys (Figure 7K) and in RCC4 cells (Supplementary Figure S3F–H). In TRACK mice, ATF4 deletion did not cause significant changes in the transcripts of enzymes in the TCA cycle. We speculate that ATF4 deletion affects the metabolites in the TCA cycle by increasing the precursors of essential co-factors (NAD^+^ and NADH) (Figure 7L), nicotinic acid (NA) and, especially, nicotinamide riboside (NR) (Figure 7M).

ATF4 deletion also affected the pentose phosphate pathway (PPP) in both RCC4 cells and TRACK mice (Supplementary Figures S3 and S4). In ccRCC, the PPP is highly upregulated to provide ribose 5-phosphate for nucleic acid synthesis and NADPH for redox homeostasis and lipid synthesis, crucial for cancer cell growth and survival [68,69]. The non-oxidative branch of this pathway, which produces erythrose-4-phosphate (E4P), is rerouted to supply the rapid anabolic needs of cancer cells [70]. Increased E4P has been reported in skin cancer [71]. In TRACK cortices, E4P is drastically increased (Supplementary Figure S4) and ATF4 deletion reduced E4P by 5-fold, suggesting that ATF4 deletion can block another energy supply route for cancer cell proliferation. However, in RCC4 cells, ATF4 deletion increased E4P (Supplementary Figure S3D,E).

## 5. Conclusions

To summarize, utilizing a combination of transcriptomics, metabolomics, and metabolite isotope tracing, we demonstrate that ATF4 deletion reduces overall glycolysis and enhances mitochondrial oxidation in ccRCC. ATF4 deletion can reprogram glucose metabolism towards more oxidative processes, providing a molecular basis for developing ccRCC therapeutics by targeting ATF4.

## Data Availability

Our mRNA-seq data are deposited into Gene Expression Omnibus (GEO), accession # GSE291049.

## Supplementary Materials

The following supporting information can be downloaded at: https://www.mdpi.com/article/10.3390/cancers18121953/s1, Figure S1: Effects of ATF4 Deletion in the Proximal Tubules of TRACK Mice on mRNAs Participating in Glycolysis; Figure S2: ATF4 Deletion in RCC4 Cells Enhances Oxidative Activities in the TCA Cycle; Figure S3: ATF4 Deletion in RCC4 Cells Alters Metabolites in the Pentose Phosphate Pathway; Figure S4: ATF4 Deletion in the Proximal Tubules Modulates Metabolites in the Pentose Phosphate Pathway in TRACK Kidneys.
