# Supplementary material for: Transcription Factor ATF4 Deletion Reprograms Glucose Metabolism in Clear Cell Renal Cell Carcinoma
Source: Cancers (Basel). 2026 Jun 16;18(12):1953. doi: 10.3390/cancers18121953 (PMC13297217; doi:10.3390/cancers18121953)
Supplement: Supplementary file 1 [file cancers-18-01953-s001.zip › cancers-4336742-supplementary.pdf]

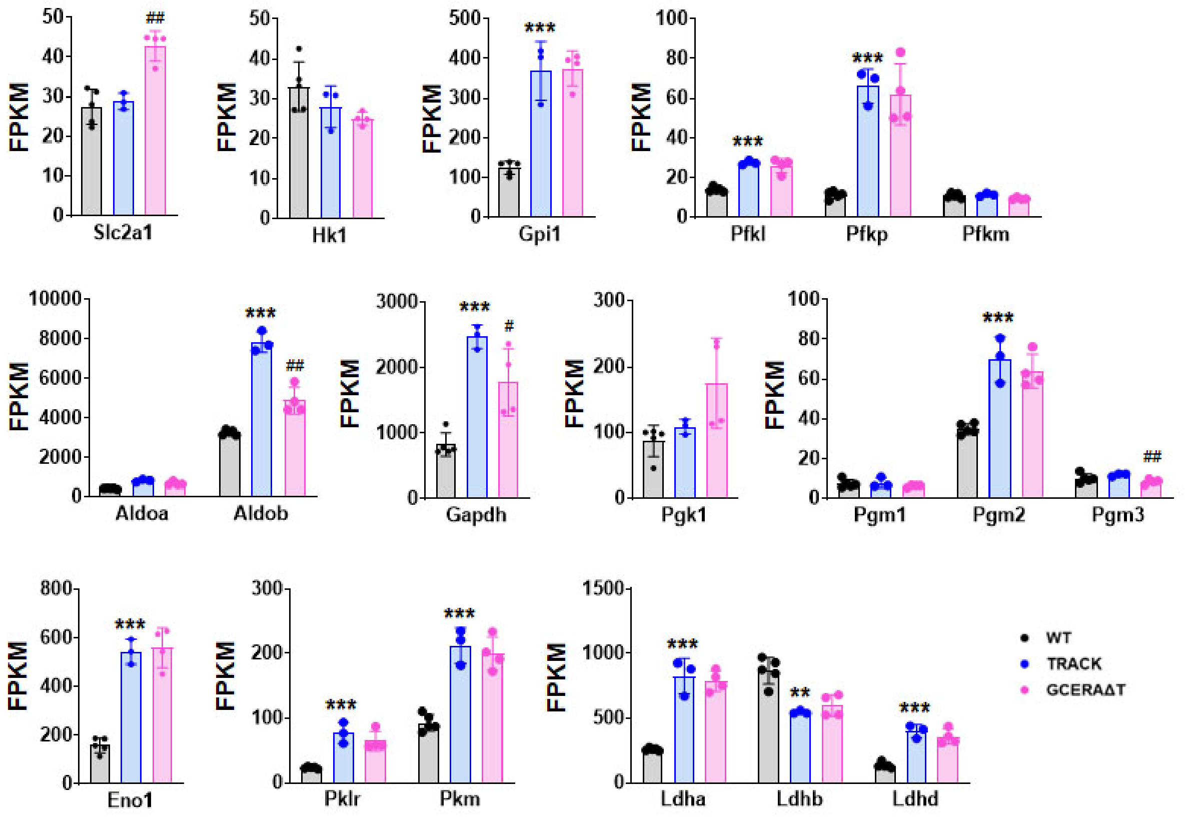

Supplementary Figure S1. Effects of ATF4 Deletion in the Proximal Tubules of TRACK Mice on mRNAs Participating in Glycolysis. For comparison between TRACK vs WT cortices, $^{**}p \leq 0.01$; $^{***}p \leq 0.001$; for comparison between GCERAΔT vs TRACK, #$p \leq 0.05$; ##$p \leq 0.01$. Slc2a1 (Glut1), Solute carrier family 2 member 1 (Glucose transporter member 1); Hk: Hexokinase; Pgi, Phosphoglucose isomerase; Pfkl, Phosphofructokinase, liver; Pfkm, Phosphofructokinase, muscle; Pfkp, Phosphofructokinase, platelet; Aldo, Aldolase; Gapdh, Glyceraldehyde-3-phosphate dehydrogenase; Pgk1, Phosphoglycerate kinase 1; Eno, Enolase; Pgm, Phosphoglucomutase; Pklr, Pyruvate kinase L/R; Pkm2, Pyruvate kinase M2; Ldha, Lactate dehydrogenase a; Ldhb, Lactate dehydrogenase b; Ldhd, Lactate dehydrogenase d.

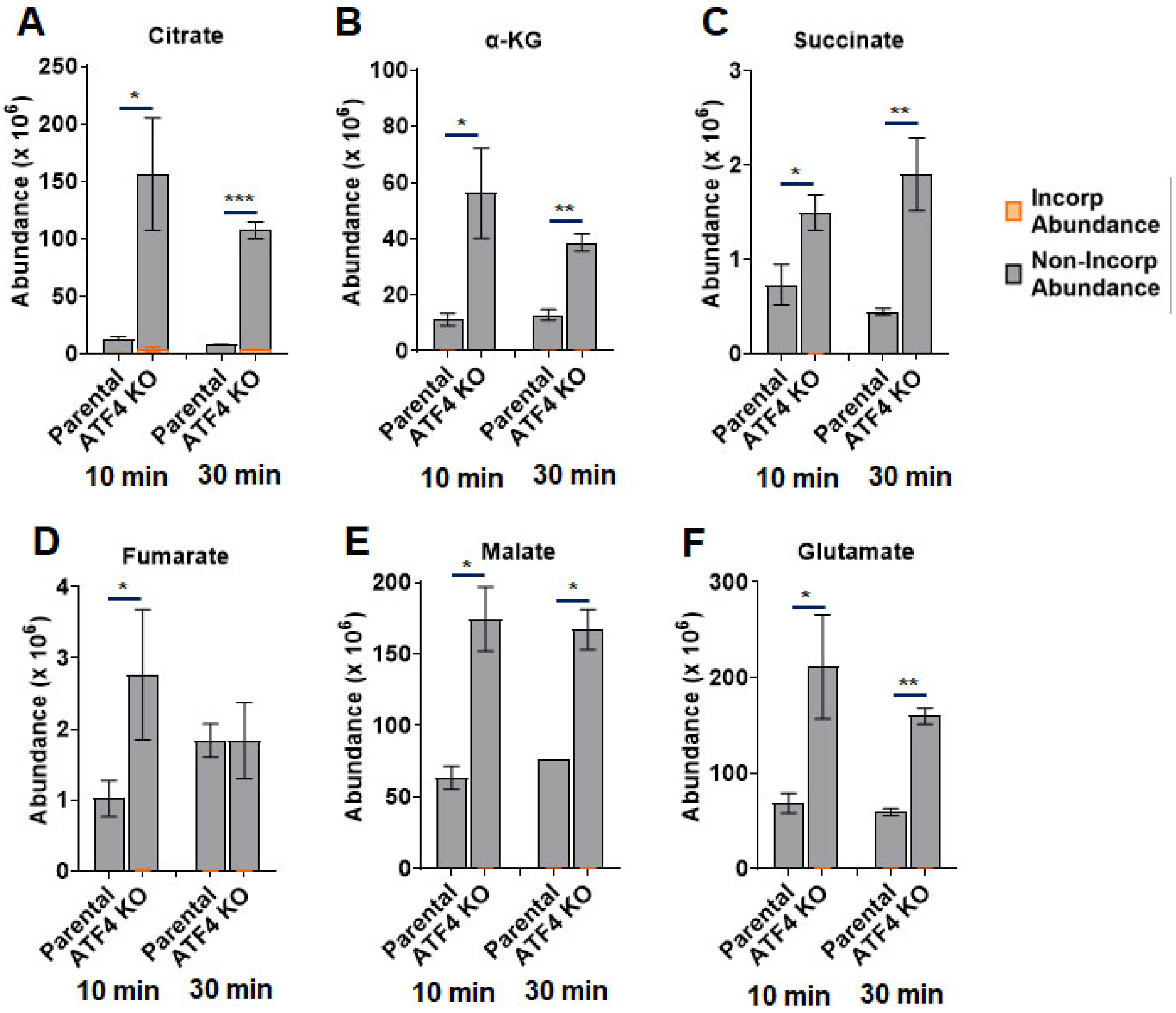

Supplementary Figure S2. ATF4 Deletion in RCC4 Cells Enhances Oxidative Activities in the TCA cycle. A-F, abundances of metabolites incorporated or non-incorporated with ^{13}C isotopes at 10 min and 30 min after cells were fed with [$U^{13}C_6$]glucose. α-KG, alpha-ketoglutarate. *$p \leq 0.05$; **$p \leq 0.01$.

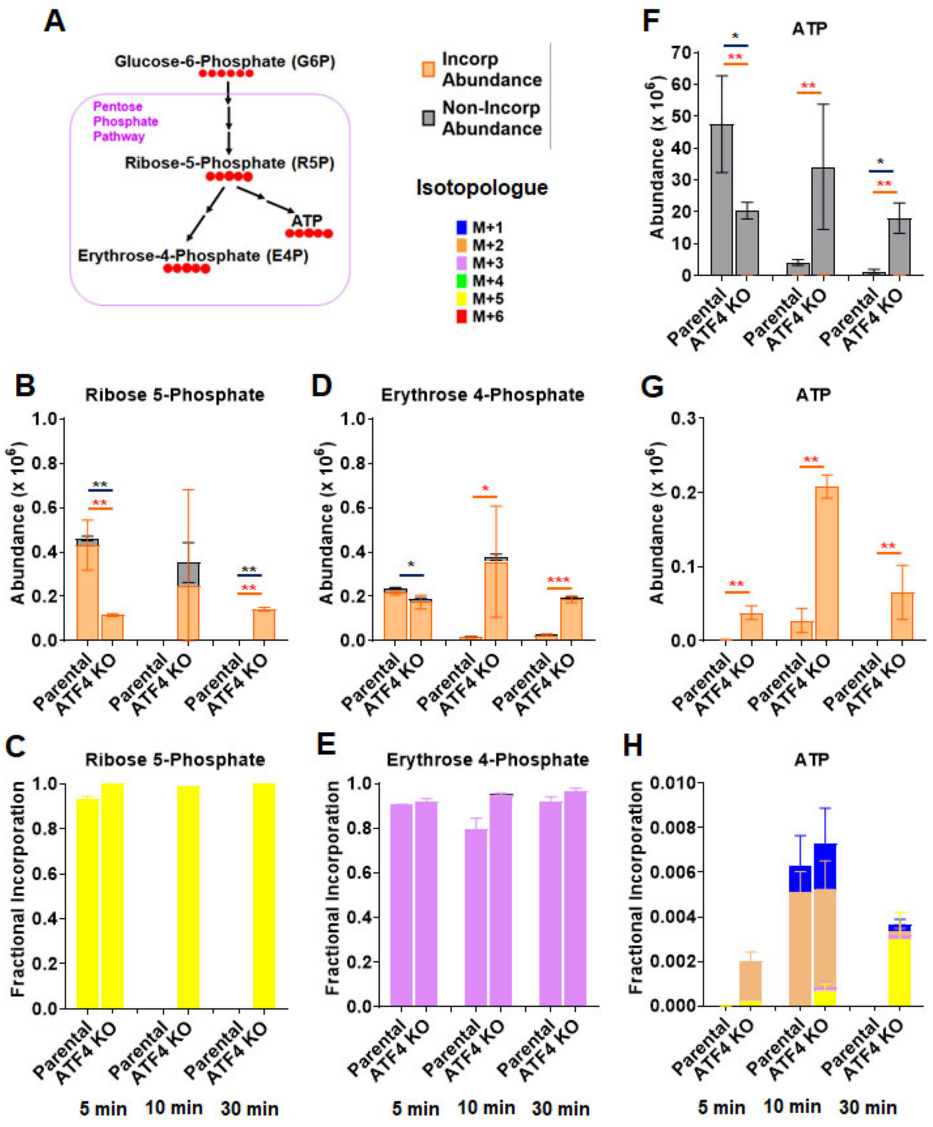

Supplementary Figure S3. ATF4 Deletion in RCC4 Cells Alters Metabolites in the Pentose Phosphate Pathway. A, diagram of pentose phosphate pathway; B-E, F and H, abundances of metabolites incorporated or non-incorporated with ^{13}C isotopes and their corresponding fractional incorporations at 5, 10 and 30 min after cells were fed with $[U^{13}C_6]$glucose; G, abundance of ATP incorporated with ^{13}C isotopes. *$p \leq 0.05$; **$p \leq 0.01$. Orange stars indicate significance for comparisons of abundances incorporated with ^{13}C isotopes between parental and ATF4KO cells; black stars indicate significance for comparisons of abundances non-incorporated with ^{13}C isotopes between parental and ATF4KO cells.

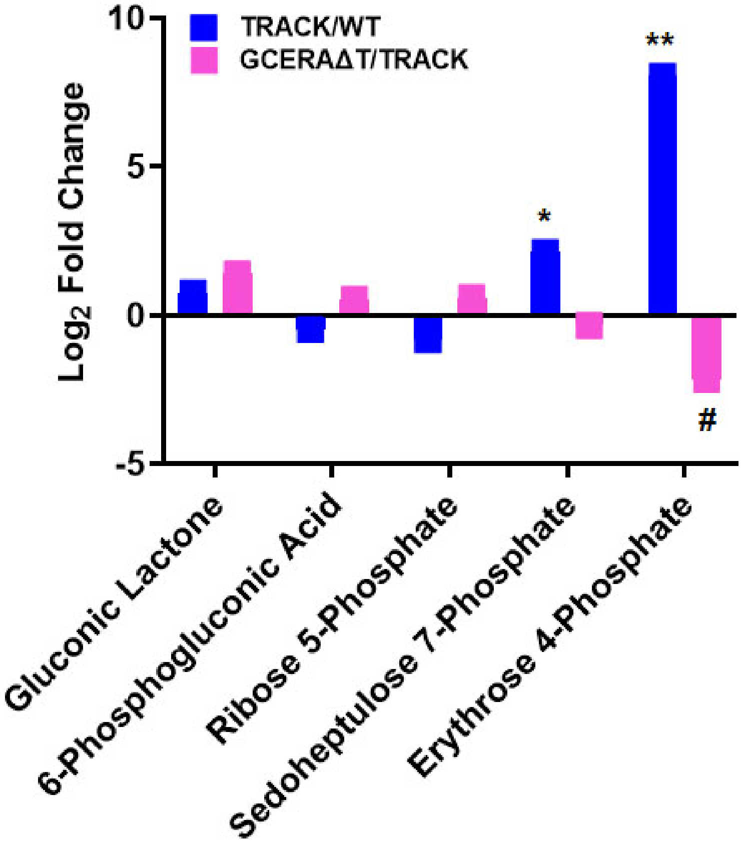

Supplementary Figure S4. ATF4 Deletion in the Proximal Tubules Modulates Metabolites in the Pentose Phosphate Pathway in TRACK kidneys. For comparison between TRACK vs WT cortices, $*p \leq 0.05$; $**p \leq 0.01$. For comparison between GCERAΔT vs TRACK, $\#p \leq 0.05$.
